# Is birth weight the major confounding factor in the study of gestational weight gain?: an observational cohort study

**DOI:** 10.1186/s12884-018-1843-9

**Published:** 2018-06-07

**Authors:** Amy C. O’Higgins, Anne Doolan, Thomas McCartan, Laura Mullaney, Clare O’Connor, Michael J. Turner

**Affiliations:** UCD Centre for Human Reproduction, Coombe Women and Infants University Hospital, Dublin 8, Ireland

**Keywords:** Gestational weight gain, Maternal obesity, Birth weight

## Abstract

**Background:**

Much interest has been focussed on both maternal obesity and gestational weight gain (GWG), particularly on their role in influencing birth weight (BW). Several large reviews have reported that excessive GWG is associated with an increase in BW^.^ However recent large, well-designed, randomized controlled trials studying interventions aimed at reducing GWG have all consistently failed to show a reduction in BW despite achieving a reduction in GWG. The aim of this longitudinal prospective study was to examine the relationship between GWG and birth weight in women where GWG and Body Mass Index (BMI) were measured accurately in a strictly standardized way.

**Methods:**

Women were enrolled at their convenience before 18 weeks gestation. Height and weight were measured accurately at the first antenatal visit and BMI calculated. Maternal weight was measured again after 37 weeks gestation. The weight of the baby was measured at birth. Relationships were tested using linear regression analysis, chi-squared tests and t-tests as appropriate.

**Results:**

Of the 522 women studied, the mean BMI was 25.3 kg/m^2^ and 15.7% were obese. The mean BW at term was 3576 g (2160–5120) and 2.7% (*n* = 14) weighed ≥4500 g. The mean GWG overall was 12.3 kg (4.6 to 28.4) and GWG decreased as BMI increased. The mean GWG was less in obese women, at 8.7 kg (− 4.6 to 23.4), compared to non-obese,13.0 kg (0.6–28.4) (*p* < 0.001). Mean BW in obese women was 3630 g vs 3565 g in non-obese (*p* = 0.27). The total GWG correlated positively with BW (*p* < 0.001). When BW was subtracted from total GWG, GWG no longer correlated with BW (*p* = 0.12).

**Conclusions:**

The positive correlation between GWG in pregnancy and BW can be accounted for by the contribution of fetal weight to GWG antenatally without a contribution from increased maternal adiposity. There was a wide range of BW irrespective of the degree of GWG and obese women had a lower GWG than non-obese women. These findings help explain why Randomized Controlled Trials (RCTs) designed to reduce GWG have failed to decrease BW and suggest there is no causative link between excessive GWG and increased BW.

## Background

Obesity is now a major health problem and is now associated with more deaths globally than malnutrition [1]. There are multiple, complex factors driving the increase in rates of both adult and childhood obesity [1]. Recently much interest has focussed on both maternal obesity and gestational weight gain (GWG), particularly on their role in influencing birth weight (BW). Several large reviews have reported that excessive GWG is associated with an increase in BW [2–5].

However, we are concerned that there are limitations to previous studies that have not been adequately addressed [6]. In particular, these studies are large epidemiological studies where maternal height and weight is self-reported and not measured, leading to the miscategorization of baseline BMI, the under-estimation of obesity rates and the over-estimation of the *impact* of obesity of *clinical* outcomes [7, 8]. Since the publication of revised guidelines on GWG by the Institute of Medicine (IOM) in the United States in 2009 [2], many studies of GWG have reported their outcomes in relation to ‘excessive’, ‘adequate’ or ‘inadequate’ GWG as defined by the IOM [9–12]. The IOM guidelines give different ranges of recommended GWG depending on a woman’s baseline BMI*. Underweight women have a recommended GWG of 12.5–18.0 kg, women of normal BMI have a recommened GWG of 11.5–16.0 kg, overweight women have a recommended GWG of 7.0–11.5 kg and obese women a recommended GWG of 5.0–9.0 kg. This terminology is a source of bias in the current literature since a GWG of 10 kg is considered ‘inadequate’ for women of a normal BMI and ‘excessive’ for obese women.* It prevents correlation of maternal and fetal outcomes with absolute GWG and makes it difficult to understand the basic physiological correlations between GWG and clincial outcomes including BW.

Recently several large, well-designed, randomised controlled trials studying interventions aimed at reducing gestational weight gain have all consistenly failed to show a reduction in BW despite achieving a reduction in GWG with their intervention [13–17]. We believe these results raise doubt as to a causal relationship between GWG and BW.

We examined the relationship between absolute GWG and BW at term in a well-characterised cohort of women where the calculation of Body Mass Index (BMI) and GWG was accurately assesed in a strictly standardized way.

## Methods

The study was undertaken between July 2012 and July 2014 at, *the Coombe Women and Infants Univeristy Hospital*, a large university teaching hospital with over 8500 deliveries per annum. The Hospital cares for women from rural and urban regions and from all socio-economic groups. The study was confined to white European women with a singleton pregnancy *to reduce confounding variables associated with differences in fat mass between women of differing ethnicities of the same BMI* [18]. Women with pre-existing diabetes mellitus were excluded.

Women were recruited at their first antenatal visit *which takes place in the Hospital.* An information leaflet was given and written informed consent obtained. The study was approved by the Hospital’s Research Ethics Committee. At recruitment all women had their pregnancy dated by ultrasound and only women < 18 weeks gestation were included. We have previously reported in a cross-sectional study that there is no significant change in mean maternal weight or body composition before this gestation [6, 19]. Maternal sociodemographic and clinical details were recorded and computerized.

Maternal height was measured at recruitment to the nearest 0.1 cm using a digital wall-mounted stadiometer (Seca, Birmingham, United Kingdom) with the woman standing in her bare feet. *Weight was measured using a digital weighing scales* (Tanita MC-180MA, Tokyo, Japan). Women were asked to empty their bladder prior to measurement *which* was performed in light clothing and bare feet [19]. BMI was categorized according to the World Health Organization [20]. Term was defined as the period after *36* completed weeks of gestation. Maternal weight measurement was measured again at a routine antenatal visit at term.

Women with a risk factor for the development of gestational diabetes mellitus (GDM) according to the national guidelines underwent screening with a 75 g oral glucose tolerance test (GTT) between 24 and 28 weeks gestation [21, 22]. The women received their clinical care from their own obstetric teams and not from the research team.

Infants were weighed within an hour of birth using a digital scales wearing only their identification band and umbilical cord clip. We defined total GWG as the measured maternal weight from 37 weeks gestation minus the measured maternal weight at the first antenatal visit. Total GWG includes both a maternal component as well as the weight of the fetus, placenta and *amniotic fluid*. We approximated the maternal component of GWG, which we called net GWG as total GWG minus BW.

Analysis was performed using Microsoft Excel (version 14.2, Microsoft, Redmond, United States of America (USA)) and SPSS (version 20.0, IBM Corp., Armok, New York, USA). *Data were tested for normality using a Shapiro-Wilk test and a normality was taken as a significance value > 0.05.* Differences between proportions were tested using chi-squared analysis, differences between means using independent samples t-test *for normally distributed data and a Mann-Whitney U test for non-normally distributed data.* Differences between continuous variables were tested using linear regression analysis *and Pearson coefficients for normally distributed data*. A *p* value < 0.05 was considered statistically significant.

## Results

Of the 1061 women initially recruited, four women had pre-existing diabetes mellitus, 15 miscarried, three women experienced preterm stillbirth, 31 delivered elsewhere, 46 had preterm live births and 440 did not return to the research centre to have a weight measurement performed after 37 weeks gestation. There were 522 women included in the final analysis. There were no differences in baseline characteristics between the 522 women included in the final analysis and those not included (Table 1). The mean gestation of the baseline weight measurement was 12.3 weeks (6.0 to 17.7, *SD 1.7*) and the mean gestation of the term weight measurement was 38.0 weeks (37.0 to 41.7, *SD 0.8*). *Of the cohort, 88% (n = 456) had the first weight measurement before 14 completed weeks of gestation and 12% (n = 66) had the first weight measurement between 14 and 18 weeks gestation*. The mean GWG was 12.3 kg (− 4.6 to 28.4, *SD 5.0*). The mean infant BW was 3576 g (2160 to 5120, *SD 448*). The range of GWG and BW at each level of maternal BMI was wide (Table 2).

**Table 1:** Characteristics of the study population compared with women who did not participate after consenting (*n* = 1061)

Characteristic	Final cohort (*n* = 522)	Women excluded (*n* = 539)	*p*
Mean maternal age (years)	30.2 (18.1–44.2)	29.9 (18.0–45.1)	0.79
Mean maternal BMI (kg/m^2^)	25.3 (16.6–48.3)	25.5 (15.5–52.4)	0.40
Nulliparas (%)	42.3 (221)	40.8 (220/539)	0.62
Obesity (%)	15.7 (82)	15.8 (85/539)	0.98
Smoking (%)	10.7 (56)	13.9 (75/539)	0.11
GDM (%)	6.5 (34)	5.6 (27/486)	0.64
Mean GWG (kg)	12. 3 (−4.6–28.4)	n/a	
Mean birth gestation (weeks)	40. 2 (37.3–42.0)	39.7 (27.0–42.4)	0.06
Mean birth weight (g)	3576 (2160–5120)	3475 (950–5120)	0.06
Macrosomia ≥4.5 kg (%)	2.7 (14)	2.1 (10/486)	0.65
Male infants (%)	49.0 (256)	50.2 (244/486)	0.71

**Table 2:** Gestational weight gain and birth weight by baseline BMI category (*n* = 522)

Baseline BMI	Mean total GWG (kg)	Mean BW (g)	Mean net GWG (kg)
Underweight (*n* = 15)	12.5 (8.3–19.4)	3266 (2670–3680)	9.2 (5.5–15.7)
Normal (*n* = 284)	13.2 (3.7–26.9)	3555 (2425–4860)	9.6 (0.6–24.1)
Overweight (*n* = 141)	12.6 (0.6–28.4)	3617 (2166–4720)	8.9 (− 2.8–24.8)
Obese I (*n* = 48)	10.3 (− 4.6–21.7)	3707 (2160–5120)	6.6 (− 7.7–18.0)
Obese II & III (*n* = 34)	6.6 (− 3.3–23.4)	3523 (2880–4580)	3.1 (−6.8–19.9)

**Fig. 1:**
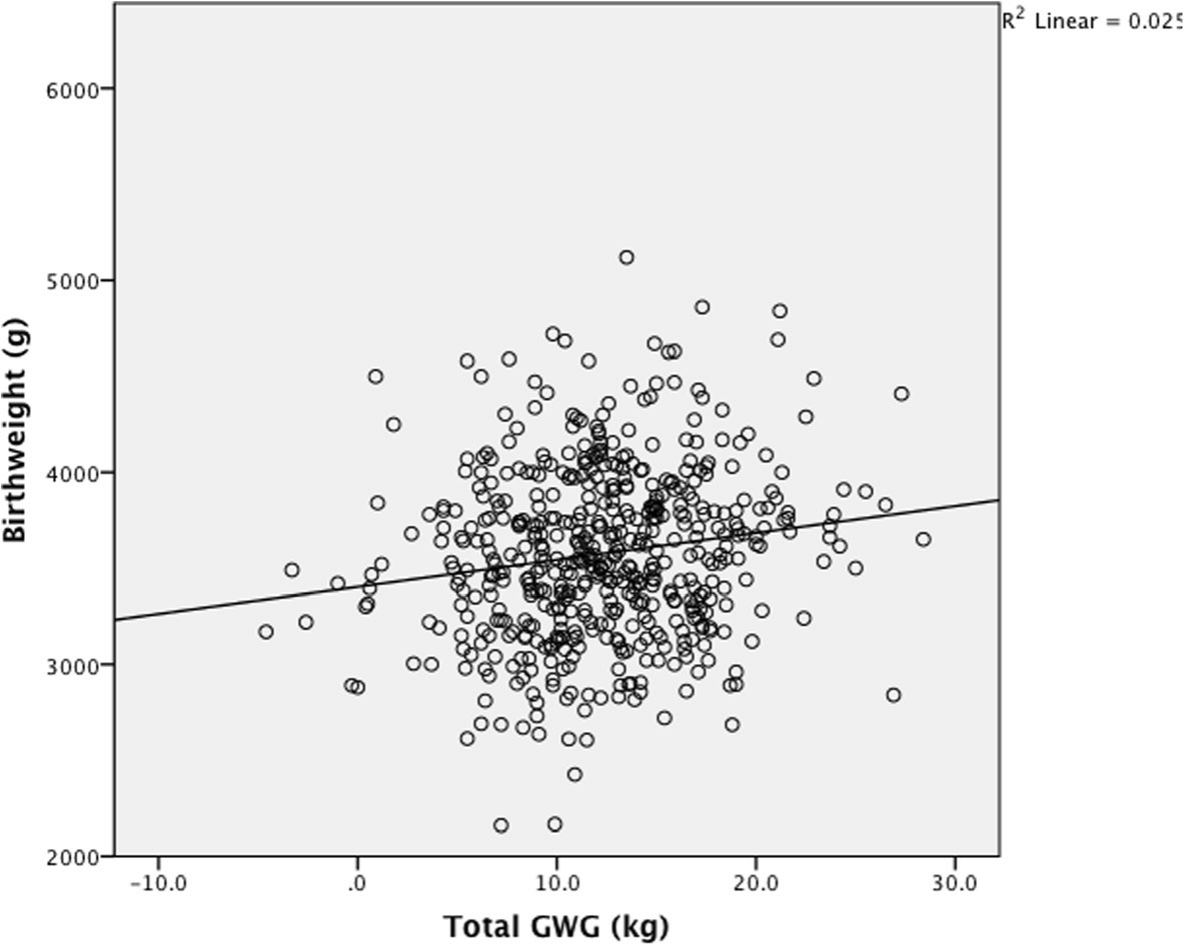
Birth weight by total gestational weight gain (*n*=522) *p*<0.001, r^2 0.025

**Fig. 2:**
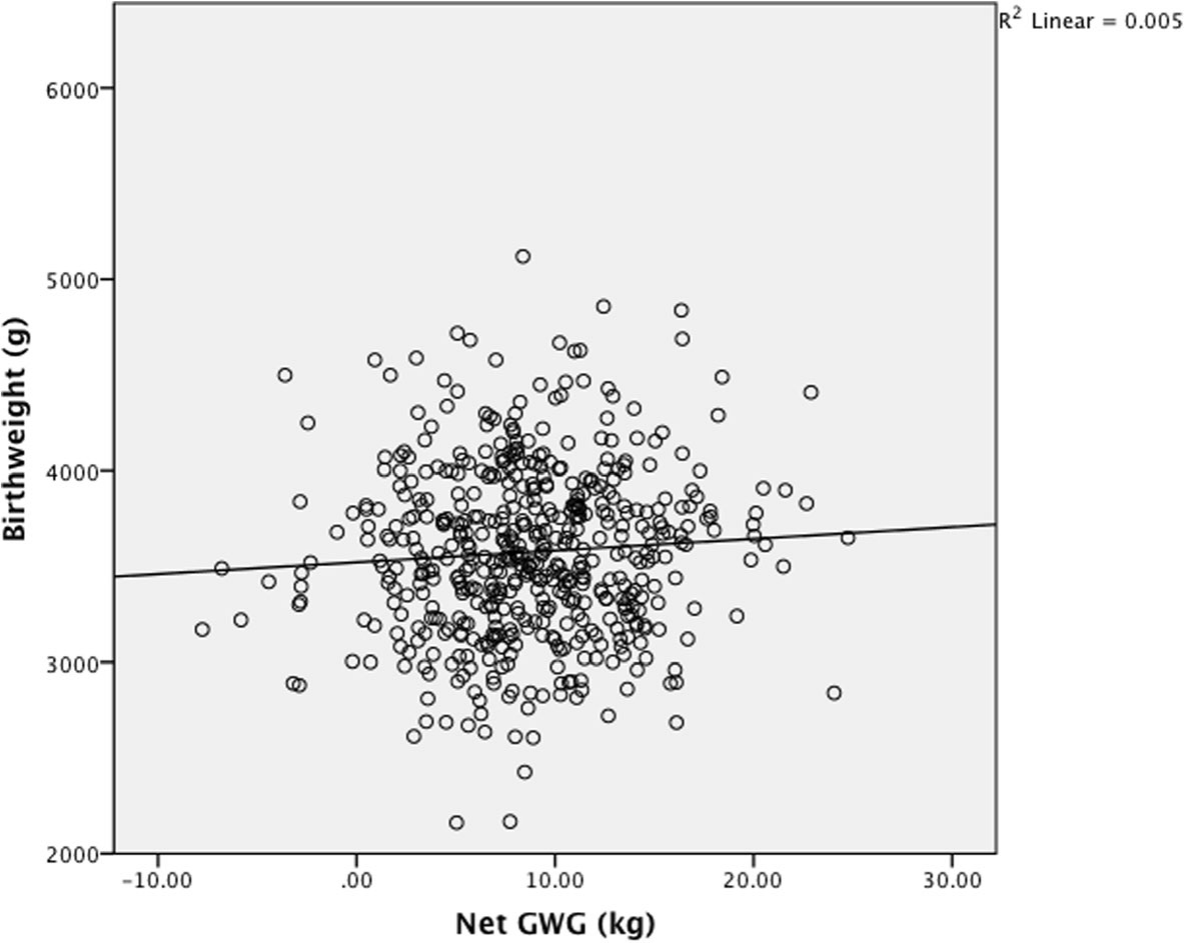
Birth weight by net gestational weight gain (*n*=522) *p*=0.12, r^2 0.005

*Maternal total GWG, net GWG and BW were all normally distributed (Shapiro-Wilk p = 0.11, 0.11, 0.40 respectively). Total GWG correlated with infant BW assessed with the Pearson coefficient (p < 0.001, r = 0.16) and on linear regression analysis (p < 0.001, r*^*2*^ *0.025) (*Fig. 1*), however when infant BW was subtracted from the total GWG, the remaining net maternal weight gain no longer correlated with infant BW assessed by Pearson coefficient (p = 0.12, r = 0.07) and by linear regression analysis (p = 0.12, r*^*2*^ *0.005)* (Fig. 2). *When booking BMI category was controlled for in the regression model the same relationship was found.* The range of BW seen at each quintile of net maternal weight gain was large. There was no difference in BW for infants born to mothers in the highest or lowest quintiles of net maternal weight gain (Table 3).

**Table 3:** Mean birth weight (g) by maternal quintile of net GWG (*n* = 522)

	Birth weight	Significance of BW difference compared to quintile 3, t-test (*p*)
Quintile 1 GWG − 7.8 to 5.0 kg (*n* = 104)	3548 (2160 to 4590)	0.45
Quintile 2 GWG 5.1 to 7.6 kg (*n* = 105)	3529 (2635 to 4720)	0.28
Quintile 3 GWG 7.7 to 9.7 kg (*n* = 104)	3596 (2166 to 5120)	n/a
Quintile 4 GWG 9.8 to 12.9 kg (*n* = 105)	3591 (2720 to 4860)	0.93
Quintile 5 GWG 13.0 to 24.8 kg (*n* = 104)	3606 (2685 to 4860)	0.88

## Discussion

In this prospective longitudinal study we found that the positive correlation between GWG in pregnancy and BW can be accounted for by the contribution of fetal weight to GWG antenatally without any contribution from increased maternal weight. Thus, epidemiological studies linking excessive GWG with increased BW or macrosomia may be statistically self-fulfilling. There was also a wide range of BW irrespective of the degree of GWG and, in the absence of any interventions, obese women had a lower GWG than non-obese women. Even when controlling for baseline BMI we found a strong relationship between total GWG and BW and no relationship between net GWG and BW. This suggests that irrespective of baseline BMI, *net* GWG does not contribute to BW. These findings help explain why RCTs designed to reduce GWG in obese women have failed to decrease BW and suggest there is no causative link between excessive GWG and increased BW.

Our study has strengths. The study population was well characterized, and both BMI and GWG were calculated using standard methodology by a single researcher. Since gestational age at delivery is one of the strongest determinants of BW our early pregnancy confirmation of gestational age by ultrasound is important. The calculation of BMI was based on the measurement of weight and height before 18 weeks gestation. We have previously directly measured maternal weight and body composition in early pregnancy and have shown that this does not alter before 18 weeks gestation [6, 19]. The calculation of GWG was also standardized.

A potential weakness of our study is that the findings may not be applicable to other populations as it was confined to white European women and the study was limited to singleton pregnancies. *Our data do not allow us to assess why such large variations in GWG were seen across all levels of maternal baseline BMI, nor what factors influence GWG. Since antenatal care was not directed by the research team, factors that may have impacted on GWG such as verbal advice given during the pregnancy were not recorded.* As this was a longitudinal study requiring repeat measurement by a single researcher over the course of pregnancy, for practical reasons, recruitment was convenient rather than consecutive.

Previous reviews, including the IOM guideline review, have acknowledged the limitations of the current evidence about GWG [2, 4, 23]. There is no standardization of definitions for GWG or fetal macrosomia. The findings are not consistent, particularly across BMI categories, and GWG is usually not corrected for BW.

Since the publication of the revised IOM guidelines, studies have reported an association between higher BW and GWG exceeding the recommendations [24–26]. Again, the measurements of gestational age, BMI and GWG were not standardized and were of variable quality. The IOM designation of “excessive” GWG is stricter for obese women, both in absolute terms and as a proportion of body weight. Therefore, comparisons between BMI groups may not be biologically valid since “excessive GWG” is defined differently at each level of BMI.

We found that even in the absence of pregnancy interventions, obese women have lower GWG than non-obese women [2, 27]. At a population level, large increases in the rates of maternal obesity have not been associated with an increase in babies born weighing > 4.5 kg or > 5.0 kg and there has not been an increase in mean BW [28, 29]. Pregnancy interventions designed to modify GWG have not been shown to change BW [12–16, 30, 31]. Furthermore, our research group has shown that offspring BW is associated with maternal fat-free mass and not maternal fat mass [32]. Thus, attempts to restrict GWG by decreasing maternal adiposity are unlikely to prevent high BW and may potentially do harm by restricting fetal nutrition.

If a woman standing on a weighing scales is given her newborn baby to hold, we would expect statistically, if powered strongly, that there would be a positive association between their combined weight and the weight of the baby she is holding. It is hardly surprising therefore, that there is a positive association between total GWG antenatally and BW at term, particularly in large epidemiological studies, since total GWG includes the weight of the baby. It would also be expected that any positive association is stronger if maternal component of GWG in lower, as is generally the case in obese women.

## Conclusions

Our findings show that BW varies widely for the same total GWG and that GWG does not correlate with BW once the weight of the baby is subtracted, do not support a causal relationship between GWG and BW. This absence of a causal relationship has also been supported by the results of the recent trials, all of which have failed to show a modification in BW despite modifying GWG [13–17].

We believe these results are important to all those involved in the care of pregnant women and their babies. This has important implications for those giving advice to pregnant women. Modification of GWG in obese women is unlikely to influence the growth of the baby. Focussing on dietary restriction, particularly in obese women who are at increased risk of micronutrient deficiency, has the potential to deprive the fetus of essential nutrients. We believe that the focus should be on helping women achieve a varied, well-balanced diet rather than on restricting weight gain. Future research on GWG needs to standardize the measurement of GWG and maternal adiposity.

## Abbreviations

BMI: Body mass index
BW: Birth weight
GDM: Gestational diabetes mellitus
GWG: Gestaional weight gain
IOM: Institute of Medicine
RCT: Randomized controlled trial
